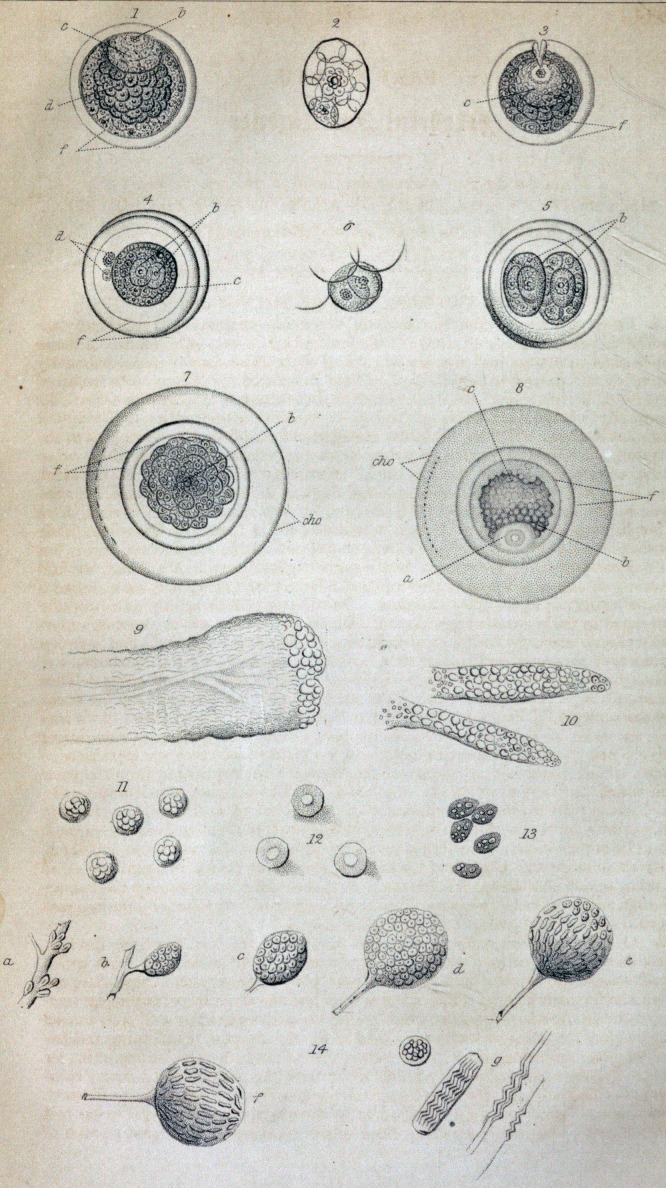# Report on the Results Obtained by the Use of the Microscope in the Study of Anatomy and Physiology

**Published:** 1843-01

**Authors:** William B. Carpenter

**Affiliations:** Lecturer on Physiology in the Bristol Medical School


### PART FOURTH.

### Medical Intelligence.

REPORT ON THE RESULTS OBTAINED BY THE USE OF THE

### MICROSCOPE IN THE STUDY OF ANATOMY AND PHYSIOLOGY.

### PART II.—*On the Origin and Functions of Cells.*

BY WILLIAM B. CARPENTER, M.D.,
Lecturer on Physiology in the Bristol Medical School.

### GENERAL FUNCTIONS OF CELLS IN VEGETABLES.

1. FROM the time when the study of vegetable anatomy was sufficiently advanced to permit the classification of the few and easily-recognized forms of tissue which exist in plants, and the knowledge of their physiology became sufficiently extensive and precise to permit their various functional operations to be referred to distinct organs, it has been evident that all those changes in which the organic life essentially consists are performed through the medium of *cells* alone, in the highest and most complex structure, as in the meanest and simplest. The functions of absorption, assimilation, fixation of carbon from the atmosphere, respiration, exhalation, secretion, and reproduction, are all effected by the one single cell, of which each individual among the humblest aerial flags, or the mucedinous fungi, consists. Even in the larger and apparently more complex structures of the sea-weeds, there is but little more separation of these functions; for in these also the whole fabric consists of cells, of which every one lives almost entirely for itself alone, and is but little dependent upon its fellows. In the highest plants, on the other hand, we find a variety of organs adapted to perform the different functions; and we also find a greater variety of elementary structure. But the parts of these organs essentially concerned in the respective functions of each are still *cells;* and the new or superadded tissues are only for the purpose of establishing that communication between organs now separated from each other, which is necessary for the continuance of their actions, and for the requisite mechanical support to the soft parts. Thus the apparatus of ducts which conveys to the leaves the sap absorbed by the roots, and the network of capillary tubes which the elaborated sap traverses in its way from the leaves to the tissues it has to nourish, cannot be regarded as themselves actively participating in the vital actions of the plant; since they are mere passive canals, which would not be needed if the leaves were less distant from the parts with which they are functionally connected. In like manner, the spiral vessels, which convey air through the interior of the plant, are mere conduits, serving to bring it into contact with tissues, which, owing to their distance from the surface, would be otherwise excluded from atmospheric influence. Lastly, the woody fibres, of which so large a proportion of the fabric of the massive trunk is composed, serve no other purpose in the vital functions than that of assisting to convey fluid along the stem and branches, (or rather, permitting its transit,) and that of affording mechanical support and protection to the softer tissues.

2. All the operations concerned in the preparation of that formative fluid, at whose expense the increase of the woody structure takes place, and from which are afterwards separated those products that give it density and durability, are performed by *cells* alone. Thus, when we analyse the structure of any organ concerned in the nutritive operations of a plant, we shall commonly find it to consist of three parts: a framework of woody tissue, for the purpose of affording mechanical support, and of assisting in the conveyance of fluid; a vascular apparatus for the conveyance of air and watery fluids to or from the organ; and a tissue composed of cells, by which alone the functions peculiar to the organ are performed. Thus it is well known that the chief agent in the absorption of the fluid in contact with the roots, is neither the woody fibre which constitutes the largest portion of the fabric, nor the vessels they contain, but the succulent cellular tissue at the growing extremities of their ultimate ramifications. In like manner, the fixation of carbon from the atmosphere, and the exhalation of superfluous fluid, which are the special functions of the leaves, are performed, not by their framework of woody fibre and vessels, but by their cellular parenchyma. The same may be said of the tissue concerned in secretion; which consists of cells that have the property of separating certain ingredients of the sap elaborated by the leaves; these they either store up in their own cavities, or set free again, probably by the rupture of the secreting cell.

3. Thus all the organic functions of the highest vegetable are really performed by cells alone. It is by those constituting the green parenchyma of the leaves, that the most important of these functions are effected; for in them the crude watery sap which ascends from the roots, parts with a large quantity of its fluid, and receives an additional amount of carbon, detached by some very powerful agency that is at present quite incomprehensible, from the oxygen with which it is united by the most powerful chemical affinity in the carbonic acid of the atmosphere. From these elements are produced all the varieties of organic compounds, so numerous and diversified in the vegetable kingdom, and so important as furnishing the materials for animal as well as vegetable organization. The descending or elaborated sap is a formative fluid, ready to be converted into solid tissue. Though chiefly composed of a mucilaginous saccharine substance, yet it is evidently very far from being a mere solution of gum and sugar; since it is already prepared to exhibit vital properties altogether unlike those which such a solution would possess. When it is examined with the microscope, it is seen to contain multitudes of minute granules; and these, aggregating together, become the nuclei from which cells are formed. The contact of a living tissue appears to be the only condition requisite for the formation of such cells, and for their admittance into the general structure by forming communications with the parts already existing. The process may be well observed in the ovule, when being prepared for fecundation; for its cavity is filled with newly-forming cells, developed in the midst of the starchy albumen. In general, the duration of life in these cells is very brief; for in those species in which the mature seed has no separate albumen, they dissolve away in proportion as the embryo absorbs the starchy matter in the midst of which they are developed; and in those in which this absorption does not take place until the period of germination, several generations of cells are produced and disappear, previously to the formation of those which constitute the tissue of the albumen.

4. The conversion of the formative fluid into organized tissue, by the development of cells from the granular nuclei it contains, may also be observed in the cambium or glutinous sap intervening between the bark and wood,—the place in which the new layers of bark and wood are to be produced. Of the mode in which the first cells are produced in the formative fluid, according to the observations of Schleiden, an account has been given in a former volume (vol. IX. p. 499); and little need be here added to it, except a doubt whether the formation of a distinct nucleus or cytoblast is in all instances necessary, or whether a cell may not be formed by the simple coalescence of granules. That the function of the cytoblast is not restricted (as supposed by Schleiden) to the production of the first or primordial cell, will appear from observations to be presently adduced (§ 12.); when these are taken in connexion with the fact, observed by myself, as well as by several other microscopists, that the nucleus of vegetable cells is *not* absorbed in a great number of the cases in which it seems to be so, but either remains adherent to the wall of the cell (though not easily seen on account of its transparency), or separates into its component granules which become the germs of new cells, or contributes to the formation of the spinal fibrillæ, and other secondary products which appear at a subsequent time within the parent-cell.

5. Not only are the proper organic functions of plants thus dependent upon the agency of cells; but their reproduction is likewise. The author has elsewhere*

* Principles of General and Comparative Physiology, 2d edition, §§ 592-601.

explained in full his conclusions respecting the essential nature of this process, derived from an analysis of the operations concerned in it, in all classes of vegetables; and a very brief account of it will therefore here suffice. In all plants there exist certain cells, which contain granules that have the power of developing themselves into new cells, when altogether separated from the parent structure. In the higher tribes, these reproductive cells are very distinct from the rest, and are known as pollen-grains among the Phanerogamia, and as spores in the Cryptogamia. In the lowest tribes of the latter, where each individual consists but of a single cell, or of an aggregation of a few having similar properties, that cell has the same power of reproduction. Here, therefore, we meet with the simplest phase of the process. The parent-cell, arrived at maturity by the exercise of its organic functions, bursts, and liberates its contained granules. These, at once thrown upon their own resources, and entirely dependent for their nutrition on the surrounding elements, develope themselves into new cells, which repeat the life of their original. Such is the whole history of the reproduction in the inferior Cryptogamia. In the higher tribes of that division, the reproductive cell or spore does not burst; but the first cells of the new structure are developed within it; and these gradually extend, by a similar process of multiplication, into that primary leaf-like expansion, which is the first-formed structure in all plants. Here, it may be surmised, the continued vital operation of the parent-cell aids its contained germs in their early development, elaborating their food from the surrounding elements, until they are able to obtain it for themselves.

6. But in the Phanerogamia a more complex apparatus is superadded for this purpose; the ovule being adapted for the reception of the germs prepared by the pollen-grain, and conveyed into it by an extension of the pollen-tube; and being obviously intended to supply these with nourishment of a highly-elaborated kind, up to a comparatively late period of their development,—that at which the true leaves are unfolded, and the permanent roots embedded in the soil. During the whole of the early life of the embryo of the highest plant, its tissue consists of cells alone. The structure first formed is a leaf-like expansion (the single or double cotyledon) closely resembling that which constitutes the permanent frond of the inferior cryptogamia: this absorbs the nutriment prepared by the parent, and brought in contact with its surface; and its cells effect a still further change in it, by which it is made ready for the nutrition of the embryo that is to be developed from its centre.

7. It is interesting to observe that in this, the earliest phase of the embryonic life of the plant, there is a manifestation of that arrangement of the absorbent apparatus, which is characteristic of the vegetable kingdom in general, that, namely, which is adapted for absorption by the *external* surface. The cells of the cotyledon which is being developed in the seed of the flowering-plant, are spread forth in contact with the starchy fluid which they are destined to absorb; just as are those of the frond of the sea-weed in contact with the water it inhabits. It is only when the advancing development of the embryo requires a provision for the conveyance to it of the nourishment thus obtained, that we find vessels developed in its tissue; and it is only when its increasing size renders necessary some provision for additional strength, that woody fibre is generated.

### GENERAL FUNCTIONS OF CELLS IN ANIMALS.

8. Until a comparatively recent period, nothing has been known of the phenomena of *cell-life* in animals. Although many isolated facts had been ascertained by Purkinje, Valentin, Henle, Müller, Wagner, Turpin, and others, in regard to the existence of nucleated cells in the solids and fluids of the body, the production of these cells from pre-existent nuclei, and the development of new cells within those of a preceding generation, it was Schwann who first gave expression to the important generalization, that nucleated cells are the basis of *all* animal as well as vegetable structures; a doctrine more fruitful, perhaps, in novel results and widely-extended applications, than any other in modern physiology. For whilst some of the followers of Schwann, in the same line of inquiry, have shown that certain limitations are necessary, a few of the tissues being produced more directly, by the simple consolidation of the fluid plasma into fibrillæ and membranous lamellæ, and some (as would appear from Henle's observations) being formed by the coalescence of the elements of cells, whose development into cells has been arrested; other observers have given an extension to Schwann's doctrines that he could not have himself anticipated. For not only does it now appear that nearly all the animal tissues, however great the alterations they may have undergone in structure and properties, have their immediate origin in cells; but that *in animals as in plants, all the changes in which organic life essentially consists are performed by cells, scarcely distinguishable from each other by any well-marked characters.*

9: The author is not aware that this proposition has been yet stated in so general a form. In fact, many of the data upon which it is founded are but of very recent discovery: and it is the object of the present report to bring them systematically together, and to show their bearing on each other. It may be necessary to explain briefly the meaning and application of this statement before proceeding to the demonstration of it. The purely *animal* functions, those of the nervous and muscular systems, are not included in it; nor are those of a merely physical character, such as the movement of fluids through canals, or the resistance and support afforded by the solid and elastic tissues. These last, as in vegetables, may be regarded as *addenda* for the purpose of supplying the conditions necessary for those really vital operations in which organic life essentially consists. We know of no animal so simple as the lowest cryptogamic plant; but there is reason to believe that there are many in which no vessels exist, their tissue being everywhere in near contact with the nutrient fluid, and absorbing directly from it; and it is certain that there are many in which a few scattered muscular fibres and nervous filaments constitute the only departure from the general type of *cellular** tissue. Here, then, there is no difficulty in understanding that all the functions of organic life, absorption, assimilation, nutrition, respiration, secretion, and reproduction, must be performed by cells. Again, in the early condition of the embryo, which is at first nothing more than a mass of cells, precisely the same holds good. For some time, its life is entirely vegetative; it absorbs its nutriment by cells spread over the yelk; and this nutriment is at first applied solely to the development of new cells, some of which gradually undergo metamorphosis into other tissues. But the same will be found true of this function in the adult state of the highest animal; for nutritive absorption is in it also performed by cells, which appear destined to this function alone. In like manner it will appear that another set of cells have for their office the assimilation of the nutriment, that is, the preparation of it for entering into the composition of the living organized body. Further, it seems certain that the first development of nearly all the tissues takes place from cells, which are produced at the expense of this assimilated nutriment. Again, the separation from the circulating fluid of these products which are to be cast off from it, is also accomplished by cells. (With regard to the simple exhalation of fluid, it may be remarked that this, like imbibition, is a physical function, dependent upon the permeability of membrane; and that the vital action of cells is therefore not necessary for it. The same may perhaps be said of respiration; but we shall find that in this the action of cells is concerned. § 38.) Lastly in regard to reproduction, it appears that the essential part of this process consists, among animals as among plants, of the multiplication of cells under peculiar conditions. These various processes will now be severally considered in detail. It will be desirable, however, to examine in the first instance into the history of the development of cells in the animal; a subject which is so closely connected with the function of reproduction, that the latter may be considered most conveniently at the same time.

* Here and elsewhere in this Report, the term *cellular* tissue will be applied to the structure properly deserving that name, from its being composed of distinct cells or vesicles, like the parenchyma of plants. That which has been ordinarily termed cellular tissue in animals is much better named fibro-cellular tissue.

ORIGIN AND DEVELOPMENT OF CELLS IN ANIMALS.

10. The original statements of Schwann upon this subject (see vol. IX., pp. 505-26), based as they were upon those of Schleiden as to the history of the development of cells in plants, have been found to require certain modifications, to render them conformable to the results obtained by the researches of their numerous followers. The most important of these modifications relates to the development of cells *within* others previously existing, a mode of production which, though very frequent if not universal in vegetables, was considered by Schwann to be of comparatively rare occurrence in animals. The usual place of their formation, according to him, is the exterior of the old ones, and the space intervening between them (loc. cit. p. 522). The doctrine of the development of cells within cells involves also a new view of the constitution and functions of the nucleus. This was considered by Schwann and Schleiden to have performed its office, when the development of the cell originating from it is completed. By Schleiden, the nucleus of vegetable cells was stated to be usually reabsorbed, especially when the cell is destined to undergo still further development; though in some particular tribes of plants it is persistent during the whole life of the cell. (p. 500.) In the tissues of animals, on the other hand, the persistence of the nucleus was generally remarked by Schwann; but he scarcely records any facts which indicate that he regards its function as prolonged beyond the completion of the primordial cell. It is to Dr. Barry that we are indebted for the correction of this important error, for such I believe it is now generally admitted to be; by showing that the function of the nucleus has reference at least as much to the secondary cells, or other new structures, which are to be developed within the parent-cell, as to the first production of the latter. This was one of the novel results of Dr. Barry's embryological researches, the third series of which is devoted to an elucidation of the earliest changes that occur in the mammiferous ovum, previously and subsequently to fecundation; and as these observations not only afford the best illustration of Dr. Barry's views on the development of cells in general, but present one of the most interesting examples of that *cellular life* which it is the object of the present report to bring into notice, we shall give them our first consideration.

11. Dr. Barry's Embryological Researches. (Third Series, Philosophical Transactions, 1840.) That our readers may understand the nature and importance of Dr. Barry's later contributions to embryological science, we would refer them to an article at the commencement of our Ninth volume, which contains an account of his first memoir, and of the general state of knowledge on the subject, up to the year 1840. We need only now allude, therefore, to the discovery of the *germinal vesicle* by Purkinje, originally in the bird's egg; the universality of the existence of which, in the ova of all animals, has now been satisfactorily demonstrated by the combined labours of Baer, Coste, Valentin, Wagner, and Wharton Jones: as also to that of the *germinal spot*, which was discovered contemporaneously by Wagner and Wharton Jones in the germinal vesicle of the mammiferous ovum, but in addition traced by the former throughout the whole series of animals. By Schwann it was maintained that the germinal vesicle, being the part of the ovum first formed, was to be considered as its nucleus; and that the germinal spot is to be regarded as the *nucleolus*. In regard to the destination of the germinal vesicle, no more definite statement had been given, than that it burst or dissolved away; and that the *cicatricula* occupied its place, as if this originated in its *debris*. Nothing certain or tangible was known as to the nature of this change; still less was it imagined that the cicatricula is a mass of cells, developed from the germinal spot, within the germinal vesicle, by a process which is probably to be regarded as a type of the most essential phenomena of nutrition.

12. *Preliminary changes in the Germinal Vesicle.* Dr. Barry commences by stating that the germinal vesicle does not burst, dissolve away, or become flattened, on or before the fecundation of the ovum, as previously supposed; but that it ceases to be pellucid, in consequence of the development of new cells within it; which new cells become filled with the foundations of other cells, so that the whole is rendered nearly opaque. These new cells originate, when the ovulum is being prepared for fecundation, in the margin of the germinal spot; the granules of which enlarge, so as at first to exhibit something of a warty appearance, and afterwards to form a complete annulus of small cells. A dark central point had been previously noticed by Wagner and others, as occasionally existing in the germinal spot; and Dr. Barry states that this point always makes its appearance at a certain period, that in enlarging it resembles a dark globule or ring, and that it is caused by the existence of a space filled with fluid, which is exceedingly pellucid. The formation of new cells within the germinal vesicle makes it evident that this is itself a cell and not a nucleus; the whole structure of the germinal spot is conformable to that of the nucleus or cytoblast of other cells; and it is equally obvious that the dark central point is identical with that which has been described by Schleiden and Schwann as the nucleolus. So far, however, from regarding this nucleolus as the point around which the nucleus is at first formed, Dr. Barry appears to have satisfactorily proved that it is a transitory appearance in the centre of the nucleus, indicating that this is resolving itself at its margin into new cells. Whilst the margin of the germinal spot, now converted into a ring, is thus becoming developed into new cells, a finely granular appearance is seen in the part nearer the centre, and immediately surrounding the central pellucid space; these granules undergo a gradual enlargement, and are converted into a second annulus of cells, pushing out (as it were) that previously formed, the cells of which also are enlarging in the mean time. In the interior of this annulus, another is next formed by the same process; and in this manner the whole germinal vesicle becomes filled with cells, which are arranged in concentric rings, the cells of the outer ring being the oldest and largest, those of the ring within this being next in size, and a gradual diminution taking place in each as it is nearer the centre, where we still see the pellucid space, surrounded by granules. This condition, which indicates that the germinal vesicle is prepared for fecundation, is represented in Fig. 1; in which the germinal vesicle, marked *c*, is seen to be full of cells in various stages of development; and the yelk also is perceived to contain numerous cells, each having a central nucleus, round which new cells are being formed by a similar process. As the foundations of new cells successively make their appearance in the interior of the altered germinal spot, the pellucid central space in the latter presents changes in its size, and is sometimes scarcely or not at all to be distinguished; whence it is concluded by Dr. Barry that the foundations of the new cells have their origin in the pellucid fluid of that central space. The same remark applies to nuclei in general; and serves to explain fully the varying appearance of the nucleoli observed by Schleiden and Schwann. Each of the incipient cells within the germinal vesicle presents, with more or less distinctness, an interior aspect, which indicates the operation there of a process essentially the same as that which has taken place within the parent cell. This is even more evident in the cells of the substance surrounding the germinal vesicle at a later period; and the representation given of these by Dr. Barry may probably be considered as an equally faithful portrait of what takes place in all cells undergoing the same change. (Fig. 2.)

13. *Act of Fecundation.* At the period when this preparatory change is taking place in the germinal vesicle, it is in close contact with the *zona pellucida* (*f*, fig. 1), the membrane which surrounds the yelk and takes the place of the *membrana vitelli* of oviparous ova. During the development of cells in its interior, it presents rather a lenticular form; and *that* one of its flattened faces, in which the spot with its central pellucid space appears, is applied to an attenuated portion of the zona pellucida, which soon becomes an orifice. This change is of a very remarkable character; and to Dr. Barry, so far as we are aware, is due the whole merit of discovering it. We formerly quoted (vol. IX., p. 12,) his account of the mode in which the ovum, as it becomes mature, is carried against that portion of the ovisac which is nearest the surface of the ovary; the ovisac itself is forced in the same direction; all the investments of the ovum are gradually thinned, and at last give way, so as to allow the access to it of any agent on the exterior of the ovary, before the ovum is itself extruded. We now learn that the exterior membrane of the ovum itself undergoes the same change, so as to subject the germinal vesicle to the fecundating influence, whatever may be its nature. (Fig. 3.) The remarkable coincidence of all these alterations, which seem to have for their object the opening of a direct passage to the pellucid central spot on one side of the germinal vesicle, seems to leave no room for doubt that *this* is the point of fecundation; and further evidence that such is the case is apparent in the subsequent history. For, soon after the act of fecundation has been accomplished, the germinal vesicle regains its globular form, and returns to the centre of the ovum; the fissure in the zona pellucida at the same time closes up; and there speedily appear, in the pellucid central spot, *two new cells*, "having essentially the same form and general appearance as the cells of other parts of the ovum; but in general attaining a more considerable size, and being objects of much greater importance." (Fig. 4.) These two cells undergo rapid enlargement, and at last nearly fill the germinal vesicle. (Fig. 5.) Their increase takes place at the expense of the cells which previously occupied its cavity; for these gradually disappear by liquefaction; the outer layers, which were the first produced, being the first to undergo this change. The cells of the yelk undergo liquefaction during the same period, and have completely disappeared by the time that the germinal vesicle is filled by the "twin cells."

14. The only intimation of Dr. Barry's views on the nature of the act of fecundation, is contained in the following statement: "On one occasion, in an ovum of five hours and a quarter, I saw in the orifice of the membrane *f* (fig. 3), an object very much resembling a spermatozoon which had increased in size. Its large extremity was directed towards the interior of the ovum." In a note, Dr. Barry adds—"I am not prepared to say that this was certainly a spermatozoon; but it seems proper to record the observation." The caution which Dr. Barry here manifests, in regard to an observation of so much interest, speaks much in favour of the validity of his other statements; more especially as the inherent probability of the correctness of his idea was previously so strong. For in the lower oviparous vertebrata, the spermatic fluid is directly applied to the ova; and spermatozoa had been seen on the surface of the ovarium by Bischoff, as well as by Dr. Barry himself. It was obvious that some object was to be answered by their contact with the ova; and it seemed a legitimate influence, from the facts already stated, that this purpose is the exertion of some immediate influence on the pellucid spot of the germinal vesicle. It is highly satisfactory, however, to find Dr. Barry's statements completely confirmed by the observation of Dr. A. Farre; who is understood to have distinctly seen the spermatozoa of the earth-worm inserted into the ova, and to have exhibited them to other physiologists.

15. In order that the nature of the act of fecundation may be rightly understood, it will be desirable to digress a little, to inquire into the real character of the spermatozoa. For a long period, as is well known, these bodies have enjoyed the reputation of spermatic *animalcules;* and the imagination of some microscopists, filling up the deficiencies of their imperfect instruments, gave origin to descriptions of their various parts—head, eyes, mouth, anus, limbs, &c.—which are now generally acknowledged to have no existence. Moreover, several instances of active movement present themselves in vegetable as well as in animal structures, from which it would be absurd to infer the existence of an independent animality. Thus the sporules of many algæ, the ciliated epithelium-scales to be found on some part of the surfaces of almost all animals, and the blood-corpuscles of mammalia under peculiar circumstances (as Dr. Barry has stated,* and shown to the author,) will perform movements as regular, prolonged, and vigorous, as those of the spermatozoa. Since neither their structure nor their movements, therefore, can be regarded as valid indications of their nature, our chief source of guidance is the history of their development. By Wagner it has been shown that they are developed within cells; and that they originate in some manner from the granules formed by the dispersion of the nuclei of these cells. Their development seems, in

* On the Corpuscles of the Blood. First Series. Phil. Trans. 1840.

fact, conformable to the general history of the production of animal *fibres;* and has no correspondence whatever with the evolution of animalcules. Dr. Barry has been led by his later researches to the belief that the parent cell is in reality a metamorphosed blood-corpuscle; and that the minute discs into which the nucleus resolves itself (in the mode to be hereafter explained) form, by their linear aggregation, the filamentary bodies known as spermatozoa. If this be true, it is easy to understand how the spermatozoa may deposit, in the pellucid space on the side of the germinal vesicle (where there is probably an opening into its cavity) the nuclei which are the foundations of the two new cells that subsequently appear in it; and the act of fecundation seems thus essentially to consist of the implantation (so to speak) of the germs of one or more cells, set free by the male organs, in the nidus prepared by the female, who supplies all the materials for their development into the complete organism. How completely this view harmonises with the phenomena of vegetable reproduction, is apparent from the brief notice of the latter already given in this report. (§§ 4, 5.)

16. The mysterious process of generation has thus been unveiled almost as completely as it is likely to be; for it has been shown to be reducible to the ordinary principles which govern the nutrition of the fabric;—the difference being, that the cell-germs are not developed within the cell that produced them, but are conveyed into others, where nourishment is prepared for them by a different set of organs, which usually belong in animals to a different being.

17. *Development of the Embryo.* We return to the history of the ovum, subsequently to the complete development of the "twin cells," which now fill the entire cavity of the germinal vesicle. The membranous wall of the germinal vesicle, now distended to a large size, soon afterwards disappears by liquefaction. Each of the twin cells gives origin to two others; and each of the four thus produced, to two more; and this mode of augmentation continues, until the germ consists of a mulberry-like object, the cells of which are so numerous as not to admit of being counted. (Fig. 7.) The *mode* of development of each generation of cells is exactly the same as that by which the twin parent-cells originated in the germinal vesicle. Every cell, whatever its minuteness, if its interior can be discerned, is found filled with the foundations of new cells, into which its nucleus has been resolved. These foundations at an early period are arranged in concentric layers around a pellucid point; subsequently they are larger; at a later period, when the outer layer has partially liquefied, the inner cells have increased in size; and at a period still more advanced, each cell contains but *two*, destined to succeed the parent cell, the others having disappeared by liquefaction. (Fig. 6.)

18. The next stage of the process is the formation of a layer of cells around the yelk; this layer is an extension of the peripheral portion of the mulberry mass, which now becomes flattened against one side of the ovum. Thus originates the *germinal membrane;* which, though it does not *form* the embryo (as was once supposed) performs a very important part in its early development. At the same period a large and peculiar cell, previously in the centre of the mass, comes into view; from this cell all the permanent structure of the embryo originates. It possesses a remarkably distinct nucleus, in the form of a flattened disc, which consists (as in other cases) of minute granules, and has in its centre a transparent space filled with pellucid fluid. This embryonic cell soon partakes of the general flattening; and as its own walls and the fluids contained in it are very transparent, it forms a large *pellucid area* around the nucleus, separating it from the other cells of the germinal mass. (Fig. 8.) In the nucleus of this vesicle a distinct separation soon manifests itself between its *central* and *peripheral* portion; the latter is first developed into cells, and forms a hollow membrane, which gradually extends itself through the ovum, until its surface comes in contact with the membranous layer of cells previously formed on its surface, and thus constitutes a second or inner membranous layer. Another is subsequently produced from an inner part of the nucleus of the embryonic cell; and thus are developed the three layers of the germinal membrane, the serous or exterior, the vascular or middle, and the mucous or interior. The permanent structures of the embryo are developed from the central portion of the nucleus, which then assumes an elongated form; and it is the incipient formation of cells near the central line of this nucleus which gives rise to the appearance known as the *primitive trace*.

19. It is not our present purpose, however, to pursue the history of the evolution of the embryo, and the metamorphosis of its first-formed cells into its various kinds of tissue. Our object is to examine into the mode by which the alimentary materials prepared by the parent become subservient to its nutrition. The germinal membrane, which now completely envelopes the yelk, obviously bears a strong analogy to the cotyledonous expansion or primary frond of plants; but instead of merely spreading it out in contact with the surface of the nutritive fluid, it forms a cavity which includes it, and thus presents the earliest and simplest appearance of that organ which is peculiar to animals, and characteristic of them,—a digestive cavity or stomach, adapted to contain food, and to absorb by its *inner* surface. Now during the first period of the existence of this germinal membrane, it is composed of nothing but cells. In this respect, therefore, it corresponds with the cotyledon of plants. These cells absorb from the nutritive fluid in contact with them, the materials necessary for the development of the central embryonic structure, and they at first propagate these materials to it by their own permeability alone. At a later period, however, there is formed in the vascular layer of the germinal membrane, a system of vessels communicating with the heart of the embryo, and destined to convey to it the nutriment absorbed by the subjacent cells more rapidly and directly than it was previously supplied. Very satisfactory reasons have been given by Mr. Grainger (Baly's Müller, pp. 1557-60,) for the belief that the *vasa omphalo-mesenterica* are the real agents for introducing the nutrient matter of the yelk into the system of the embryo; and that this does not pass into the intestines by the vitelline duct, as formerly supposed. They do, in fact, correspond to the mesenteric vessels of invertebrate animals, which are distributed upon the surface of the intestine, and absorb nutriment from its cavity; since, as just shown, the yelk-bag is the temporary stomach of the embryo; and, as will presently appear, it remains as the permanent stomach of the radiated tribes. Previous to the ninth day of incubation (in the fowl's egg) a series of folds are formed by the lining membrane of the yelk-bag, which project into its cavity; these become gradually deeper and more crowded, as the vesicle diminishes in size, by the absorption of its contents. The omphalo-mesenteric vessels that ramify upon the yelk-bag send into these folds (or valvulæ conniventes) a series of inosculating loops, which immensely increase the extent of this absorbent apparatus. But these minute vessels are not in immediate contact with the yelk; for there intervenes between them a layer of nucleated cells, which is easily washed away. It was from the colour of these, communicated to the layer of vessels beneath it, that Haller termed the latter *vasa lutea*; when the layer is removed, the vessels present their usual colour. It appears from Dr. Barry's observations, that the *second* membranous layer given off from the peripheral portion of the nucleus of the embryonic cell, is by no means the final one; but that successive productions of cells continue to take place in this manner for some time. As such additional layers are not found, however, in the interior of the vascular layer, it is probable that those first produced must have disappeared; and it will be found perfectly conformable to the facts presently to be stated (§§ 21-22), to suppose that they have served to elaborate the nutriment supplied by the yelk; and that they then deliver it up to the absorbent vessels, by themselves undergoing rupture or dissolution, their place being supplied by the new layer formed in the mode just adverted to.

20. In the radiated tribes the yelk-bag remains as the permanent stomach; and whilst the inner layer of the germinal membrane forms the lining of the cavity, the outer layer composes the integument of the animal. A mouth is formed by the gradual thinning away of the membrane at one end, and tentacula sprout forth around this. It may be questioned whether any proper vessels ever exist in these animals, but there are certainly intercellular passages along which a circulation of nutritious fluid takes place. Between these passages and the digestive cavity, there is a layer of nucleated cells, closely resembling those which form the lining of the bird's egg, and doubtless having the same function, that of absorbing and partially elaborating the nutritive fluid.

NUTRITIVE ABSORPTION.

21. We pass, naturally, from the consideration of the nutrition of the embryo to that of the adult animal. It has been shown that, in the former, the absorption of the nutritious fluid by cells may be regarded as the preliminary to its introduction into the vessels. The same may be said of the adult; for, as Mr. Goodsir has recently shown,* there is a continual development of cells at the extremity of each villus, during the period when the lacteals are absorbing chyle. These cells appear to be the agents by which the *selection* of the nutritious fluid is accomplished, and by which it undergoes its first preparation for the purposes it is subsequently to fulfil. It is true that the veins also are concerned in absorption; but this is not a *selective* absorption, for they take up anything that is sufficiently soluble in the fluid they imbibe; and this imbibition has been shown to be almost certainly of a simply physical character. On the other hand, the lacteals clearly possess the power of taking up from the fluid in the digestive cavity those portions which can be rendered subservient to the nutrition of the system, and (for the most part at least) of rejecting everything else. This has always appeared a wonderful power for absorbent *vessels* to accomplish, but when the operation has been shown to be really effected by *cells*, it is seen to be exactly parallel to that which cells perform in those organisms where nothing else than cellular structure exists.

22. The following is Mr. Goodsir's account of the process. Whilst the process of digestion is going on there is an increased determination of blood to the mucous membrane of the intestinal canal, and "the minute vesicles which are dispersed among the terminal loops of the lacteals of the villus (Fig. 9) increased in size by drawing materials from the liquor sanguinis, through the coats of the capillaries, which ramify at this spot in great abundance. Whilst this increase in their capacity is in progress, the growing vesicles are continually exerting their absorbent function, and draw into their cavities that portion of the chyme in the gut which is necessary to supply materials for the chyle. When the vesicles respectively attain in succession their specific size, they burst or dissolve, their contents being cast into the tissue of the villus, as in the case of any other species of interstitial cell. The looped network of lacteals, continually exerting their peculiar function, take up the remains, and the contents of the dissolved chyle cells, as well as the other matters which have already subserved the nutrition of the villus. As long as the cavity of the gut contains chyme, the vesicles of the terminal extremity of the villi continue to develope, to absorb chyle, and to burst; and their remains and contents to be removed by the interstitial absorbent action of the lacteals. When the gut contains no more chyme, the flow of blood to the mucous membrane diminishes, the development of new vesicles ceases, the lacteals empty themselves, and the villi become flaccid. The function of the villi now ceases, till they are again roused into action by another flow of chyme along the gut." Mr. Goodsir subsequently adds, "The same function is performed, the same force is in action, and the same organ, the cell, is provided for absorption of alimentary matters in the embryo and in the adult, in the plant and in the animal. The spongioles of the root, the vesicles of the villus, the last layer of cells on the internal membrane of the included yelk, or the cells which cover the vasa lutea of the dependent yelk, and, as I have satisfied myself, the cells which cover the tufts of the placenta, are the parts of the organism in which the alimentary matters first form a part of that organism, and undergo the first steps of the organizing process."

23. It is evident, then, that there is no essential difference between the lowest cryptogamic plant and the highest animal, in regard to the act of selective absorption; for in both it is accomplished by cells, which imbibe the nutritive substance that is destined for the growth of the structure; but in the former this is applied

* See Edinb. New Philos. Journal, July, 1842; and British & For. Medical Review, vol. XIV. p. 567.

to their own development alone, whilst in the latter it is speedily given up again by them to the vessels that are to convey it to distant parts, for the renovation of which it is being prepared.

ASSIMILATION.

24. The alimentary materials taken in by the absorbent vessels are not yet in a state fit to be applied to the nutrition of the tissues; for they are in the condition of *chemical* compounds, not yet possessed (in any high degree at least) of *vital* properties. The chief constituents of the chyle, as first absorbed, are albumen and fatty matter; the former is destined to be converted into the material of the solid tissues; the latter is chiefly designed for the maintenance of the animal temperature, by the combination it is afterwards made to undergo with the oxygen introduced through the lungs. It is with the *albumen* that we are at present concerned. This principle cannot be regarded as possessed of any properties which distinguish it from ordinary chemical compounds, save its peculiarity of composition, and its tendency to putrefaction when exposed to the air. In its coagulability by heat or by acids, in its combination with alkalies as an acid, or with acids as a base, and in the absence of the power of spontaneously passing into any forms more decidedly organic than the granules which are seen when it is made to coagulate slowly, it is closely analogous to many substances which belong to the domain of inorganic chemistry. Before it is ready to be appropriated by the tissues as the material for their nutrition, it must undergo a very important change. We find in the blood another principle, *fibrin*, which differs but little from albumen in its chemical composition, but is manifestly endowed with much higher vital properties. One of the most decided indications of this difference is the tendency of fibrin to coagulate when withdrawn from the living vessels; and the appearance of distinct organization in this fibrin, especially when its concretion has taken place in contact with a living surface. (§ 41.) These facts, with many others, appear to indicate that fibrin is the material which is applied to the nutrition of the tissues; and that albumen can only be thus employed after passing through the condition of fibrin. The difference between the two is precisely analogous to the difference between the ordinary mucilage of plants, and that peculiar glutinous sap which is found wherever a formation of new tissue is taking place, and which, like the liquor sanguinis of the animal, is spontaneously coagulable, and disposed to pass of itself into a semi-organized condition.

25. The change from albumen to fibrin is, therefore, the first important step in the process of assimilation. It commences in the absorbent system; for the chyle is found to contain fibrin even before it enters the mesenteric glands; and after it has passed through them the amount of fibrin is much increased. It continues in the blood, for the quantity of fibrin is always kept up in health to a certain standard, although there must be a continual withdrawal of it for the nutritive processes, without a correspondingly regular supply; and it is found to undergo a sudden and remarkable increase under the influence of local causes. What is the cause of this change? It has been usually attributed to some influence effected upon the albuminous fluid by the living surfaces over which it is passing; and the increase in the amount of fibrin in the chyle which is specially noticed after its passage through the mesenteric glands, has been thought due to some peculiar action of the blood that may come into relation with it, through the thin walls of that capillary plexus which forms, with the convoluted lacteal tubuli, nearly the whole bulk of those glands. Perhaps, however, it may be possible to offer a more satisfactory explanation; one, at least, which shall be conformable to phenomena observed in other cases.

26. Several examples have already been mentioned in this Report of the transient existence of cells, that grow, arrive at maturity, and then disappear; apparently without performing any particular function. Thus in the albumen of the seed, this often takes place to a remarkable extent. In the yelk of the egg there is a similar transitory development of cells, of which several generations succeed each other, without any permanent structure being the result. In the germinal vesicle, again, several annuli of temporary cells are seen to occupy its cavity; and the oldest and largest of these contain another generation; yet all these disappear by liquefaction, as soon as the two permanent cells begin to be developed in the centre. Further, in the subsequent development of all the cells which are descended from these, the same process is repeated, a great number of cells being produced, only to liquefy again as soon as the two central cells make their appearance. Now is it to be supposed that all this *cell-life* comes into existence without some decided purpose? I think the physiologist would not be justified in assenting to such an idea, even if he could assign no obvious reason for the process. But if an object can be assigned, which is conformable to what we elsewhere know of the operations of cells, it may claim to be received as a sufficient explanation, until some better one can be offered. Such an explanation may, I think, be deduced from the foregoing facts, and others of like nature.

27. It has been seen that the first union of the inorganic elements into the simplest proximate principles, is effected by the *cell-life* of plants. The change of these principles into the peculiar compounds which form the characteristic secretions of plants, is another result of their cell-life. And the elaboration of those azotized principles which do not enter into the composition of the vegetable tissues, but which are prepared by them for the nutrition of animals, is another result of the same operation. The change of the mere proximate principles, gum and sugar, into the organizable matter of the nutritious sap, is also partly effected by the cells of the leaves; but we may easily imagine that it is not completed by them, and that a further assimilation is requisite, before the fluid is completely prepared for entering into the composition of a permanent organized tissue. Thus, the starchy fluid which is contained in the ovule, previously to its fecundation, is probably not in the state in which it can be immediately rendered subservient to the nutrition of the embryo; and the development of successive generations of cells, which exert upon it their peculiar vitalizing influence, may be regarded as the means by which the requisite change is effected. Exactly the same may be said of the cells which are developed, in like manner, in the yelk of the egg. The albuminous matter of this is certainly not in a condition in which it can be immediately applied to the purposes of nutrition; and the conversion of it may be regarded as commencing with the development of transitory cells within its own substance, and as being completed by means of the cells forming the inner layer of the yelk-bag. A similar purpose is probably answered by the transitory cells developed within the germinal vesicle, and by those which appear in like manner in the "twin cells," and their descendants. The nutrient materials, which serve for the production of these transitory cells, may not be applicable to cells of permanent character, until it has passed through the former, and been elaborated by them. Such a view derives confirmation from the number of analogous instances, with which the animal and vegetable kingdoms present us, of the production of structures which are to have a merely temporary operation, and to be subservient to the development of permanent organs of a higher character. Now, in the early history of the embryo, as recorded by Dr. Barry, the two cells which are the last-formed product of each parent cell, are organs of the highest importance; and such a preparation may well be supposed necessary for them. It does not take place, however, in the central embryonic cell; a large part of whose nucleus is developed into cells that have a permanent place in the embryonic structure: but the exception only adds force to the general principle; for the development of this nucleus into cells takes place at the expense of nutriment supplied to it by the germinal membrane, which has now spread over the yelk, and performs the office of absorbing and preparing the materials for the development of the embryo.

28. There are other exceptions, which seem to prove the truth of the principle, though in a different mode. The multiplication of cells appears often to take place much more rapidly than by the 2-4-8 process described by Dr. Barry as occurring in the embryonic mass; but, in these instances, the cells are not destined for any but a transitory life. Thus, in the multiplication of the blood-corpuscle, *six* young cells appear to be produced from each parent. A much larger number is probably generated during the rapid growth of vegetable structures, especially those of the fungus tribe. Thus, the *bovista giganteum* has been known to increase, in a single night, from a mere point to the size of a large gourd, estimated to contain 47,000,000,000 cellules. In such a case it is difficult to suppose that any but the most rapid mode of generating cells could have been in operation; and the idea that these could not have been developed by any such elaborate process as that just alluded to, is borne out by the fact of their extremely transitory character,—the decay of such a structure being almost as rapid as its production.

29. We have thus a class of facts, which indicates that the conversion of the chemical compound into the organizable principle,—such as mucilage into elaborated sap, or albumen into fibrin,—is effected in particular situations by the vital agency of transitory cell-life; that is, by the production of cells which are not destined to form an integral part of any permanent structure, but which, after attaining a certain maturity, reproduce themselves, and disappear,—successive generations thus following one another, until the object is accomplished, after which they altogether vanish. We shall now consider another class of facts, which seems to indicate that the same change is continually being effected in the chyle, lymph, and blood of animals, as well as in the proper juice of plants; by cells, which are either carried about with it, or which are developed for the purpose in particular situations.

30. In chyle drawn from the lacteals near the intestinal tube, there is but little appearance of organization, very few chyle-corpuscles being here seen; but a large amount of very minute molecules, having apparently a fatty character, are diffused through it.* In the chyle of the mesenteric glands, the corpuscles are extremely numerous; and they are always readily seen in the chyle of the central lacteals, and of the receptaculum chyli and thoracic duct; although their number is considerably less than in chyle drawn by pricking the lacteals of the mesenteric glands. The average size of these corpuscles is about 1-4600th of an inch; but they vary from about 1-7000th to 1-2600th. They are evidently imperfectly-formed cells, which are frequently seen to contain two or three central molecules, or a granular matter, especially after they have been treated with water, and which usually exhibit the appearance of a single round nucleus when treated with dilute muriatic acid: but in the largest corpuscles, obtained from the thoracic duct, the addition of acetic acid sometimes discloses three or four central particles, similar to those which may be frequently seen by the aid of this acid in the white globules of the blood; such corpuscles may, I think, be considered as in the act of producing new cells. This idea is confirmed by the fact communicated to me by Mr. Gulliver, that the central particles of the larger chyle-globules, like the same nuclei of the colourless globules of the blood, are rather larger, more distinct, and disc-like, than the central molecules of the common-sized chyle and lymph-globules. Now the first appearance of these cells in large number is exactly coincident with the first appearance of fibrin in the chyle, to an amount sufficient to produce spontaneous coagulation; and when this fact is connected with those which have been previously stated, the inference seems very probable, that the elaboration of the fibrin is a consequence of the production of these cells, and of their vitalizing influence upon the albumen.

31. With regard to the origin of these cells, there is ample room for difference of opinion. Among the minute particles contained in the chyle of the peripheral lacteals, some appear to be (from their solubility in ether) of an oily character;†

* See Mr. Gulliver's observations on the chyle, &c. in Appendix to translation of Gerber's General Anatomy, pp. 88-94.

† That the molecular base of the chyle may be of an oily nature, there is reason to believe from Mr. Gulliver's observations (Note to Gerber, p. 56); but the remarkably uniform, grayish ground, which this base presents in the field of vision, the singular uniformity in the size of the constituent molecules, their ready disappearance on the addition of a small quantity of cold ether to the fluid chyle, and the appearance in this mixture of a fluid matter, as described by Dr. Rees, would indicate that the molecular base must be either a peculiar variety of fatty matter, or this matter in a peculiar condition,—perhaps in the form of an emulsion, so fine and impalpable that nothing similar has yet been detected in any other animal fluid. The larger, more unequal-sized, and highly refracting oily globules of the chyle are in a very different state, and so are those of milk or cream.

whilst others are albuminous. It is considered by Wagner that the latter are the germs of the chyle-cells; and I have little doubt that this idea is correct. These reproductive granules, however, must have been produced by some previously-existing cell; and their most probable origin appears to me to be the cells already mentioned as the *selectors* of the chyle at the extremities of the villi. These cells, in bursting or liquefying, yield both their fluid contents, and their reproductive granules, to the absorbent vessels; and in these vessels the chyle appears destined to undergo a gradual alteration, under the influence of that cell-life of which the foundation is laid at the first reception of the fluid into the system. The development of these cells, and the production of their peculiar effects, require a certain time; this is provided for by the delay of the chyle in the lacteal vessels. In the lower vertebrata, there are no mesenteric glands,—a circumstance which indicates that these are not an *essential* part of the absorbent system; but, in such animals, the absorbents are immensely extended in length. In the warm-blooded vertebrata, in whose conformation the principle of concentration operates to the greatest extent, we see no such prolongation; but it is provided for by the excessive convolution of the vessels in the mesenteric glands, where it seems probable that the chyle is delayed during the development of its characteristic cells.

32. Statements of a precisely similar kind may be made, regarding the lymph and its corpuscles. Reasons have been elsewhere given by the author, for regarding the lymph, not as a fluid destined to be thrown out of the system, but as the product of that secondary digestion, by which a portion of the materials, that have formed a component part of the tissues and have been set free by their disintegration, are again rendered subservient to nutrition, and are reconveyed into the current of the circulation. (See Principles of Human Physiology, §§ 465-67.) One thing appears certain,—that, with the exception of fatty matter (of which the chyle contains a large amount, whilst the lymph presents scarcely a trace of it), there is little appreciable difference between the chyle and the lymph. The latter, like the former, contains albuminous matter, part of which undergoes a gradual transformation into fibrin; and there is precisely the same evidence as in the former case, that this change is effected by the development of cells in the fluid. The idea which we should hence form, that the lymphatic glands are organs of nutrition, in which the matters passing through them are subjected to an elaboration which prepares them for the growth and maintenance of the animal structures, entirely agrees with Mr. Gulliver's observations. (See Appendix to Gerber, pp. 97-8.)

33. The continuation of this process in the blood has been regarded by Wagner, Henle, and Wharton Jones, as one of the functions (probably the chief one) of the *red* corpuscles. To this view, however, there are certain objections, which appear to the author to be of a very decided character. In the first place, there is no constant proportion between the amount of fibrin in the blood, and the number of its red corpuscles. The researches of Andral have clearly shown, that in the inflammatory condition of the blood there is a decided increase, often a most astonishing one, in the amount of the fibrin; whilst there is no corresponding augmentation in the number of red corpuscles. Indeed, this augmentation is not incompatible with a chlorotic state of the blood; the peculiar characteristic of which is a great diminution in the amount of red corpuscles. Again, in fever, the characteristic alteration in the condition of the blood appears to be an increase in the amount of red corpuscles, with a diminution in the quantity of fibrin; yet if a local inflammation should establish itself during the course of a fever, the proportion of fibrin will rise, and this without any change in the amount of corpuscles. By such alterations, the normal proportion between the quantity of fibrin and corpuscles, which may be stated as $a:b$, may be so much altered as to become $4a:b$, or $a:4b$. Can it be supposed, then, that the elaboration of the fibrin is a consequence of the action of the red corpuscles? Another important fact, having the same bearing, has been ascertained by Remak. He found that, when an animal is bled largely and repeatedly, the quantity of red corpuscles in its blood is greatly diminished, whilst the proportion of the colourless corpuscles is increased; now, as it has been ascertained by Andral's investigations that the quantity of fibrin is but little affected by bleeding, it would seem that the white rather than the red corpuscles are the agents of its elaboration.

34. Again, there appears to be a total absence in the blood (?) of invertebrated animals of any red corpuscles * resembling those of the blood of vertebrata; yet in this fluid the elaboration of albumen into fibrin must be taking place, for the nutrition of the tissues, as in the higher animals. It is true that their nutritious fluid contains globules; but these globules bear a much stronger resemblance to the corpuscles of the chyle, or to the colourless corpuscles of the blood, than they do to the red corpuscles of the vertebrata. So well marked is this resemblance, that, as Wagner † himself has suggested, the circulating fluid of the invertebrata is to be considered as rather analogous to the chyle than to the blood of vertebrated animals,—a doctrine which appears to the author extremely probable.

35. These two objections seem of themselves sufficient, in the absence of any affirmative evidence, to overthrow the hypothesis that the elaboration of fibrin is the act of the *red* corpuscles. But there is another class of blood-particles, to which increased attention has recently been directed, and in regard to whose function the very same evidence with that just produced weighs strongly in the affirmative. These are the *colourless* corpuscles or *lymph*-globules. The former term I prefer, as expressing their distinctive appearance; the latter involves the supposition that they are identical with the corpuscles of chyle and lymph, which cannot be regarded as yet demonstrated, though it appears to me highly probable. There is a remarkable uniformity in the size and appearance of these in all vertebrate animals, notwithstanding the immense difference in the size of the red corpuscles. The colourless corpuscles have not yet been observed to vary much from the diameter of 1-3000th of an inch; whilst the red corpuscles vary from less than 1-12,000th (musk-deer) to 1-337th (Proteus) of an inch. Moreover, in very young embryos of Vertebrata (as I learn from Mr. Gulliver) the white globules are nearly as numerous as the red particles. This Mr. Gulliver has often seen in fœtal deer, about an inch and a half long. In a still smaller fœtus the blood was pale from the preponderance of the white globules; and in such embryos the coloured corpuscles have a very distinct pale globular nucleus. It is therefore a fact of much interest, that, even in the mammiferous embryo, at the period when growth is most rapid, the blood has a strong analogy to that of the invertebrata. It is then, too, most analogous to chyle; since it consists of the fluid elaborated from the organizable matter supplied by the parent, and directly introduced into the current of the circulation. The function of the placenta is double; for it is at the same time the medium of introducing into the circulating fluid of the fœtus the alimentary materials supplied by the parent; and of aerating the fluid which has traversed the fœtal system. It is not until the lungs and digestive apparatus of the embryo have commenced their independent operation, that the distinction between its blood and its chyle becomes manifest. We should expect to find in the blood of the fœtus, therefore, up to the time of birth, a large proportion of white corpuscles; and this circumstance has been noticed by Dr. Barry (and communicated to me by him) with regard to the blood of the umbilical vessels of a placenta which he had the opportunity of examining very shortly after its expulsion. The fact was regarded by Dr Barry as indicating that the production of the colourless corpuscles of the blood is independent of the chyle-globules; but I am disposed to interpret it in exactly the opposite manner; since, as I have shown, the placental blood has considerable analogy to the circulating fluid of the invertebrata, which have no lacteals, and may be considered, therefore, as partly representing the chyle of the adult vertebrated animal. The colourless corpuscles are the kind of globules *most universally present* in the circulating fluid; for whilst the red particles are confined to vertebrata, the former are found in the circulating fluid of all animals, even where there are no distinct vessels.

36. A very interesting observation has recently been published by Mr. Addison,

* The red-blooded annelida do not form an exception to this rule. The colouring principle of their circulating fluid exists in its *plasma*, not in globules.

† Physiology, translated by Willis, p. 278, et seq.

(Prov. Med. and Surg. Journ. Aug. 20, 1842) which appears to the author to throw great light on the much-disputed question of the digestive apparatus of the polygastric animalcules. The numerous globular particles generally to be seen in their transparent bodies, but becoming more numerous and distinct when the animalcules have been recently feeding, are regarded by Ehrenberg as stomachs, opening out of an intestinal tube. This account of them has been objected to by other observers, on the ground, that these particles are seen to undergo a regular movement, as if they were floating in the midst of a fluid filling the general interior cavity of the body, and that they are sometimes discharged through the anal orifice. Of the validity of this objection, the author's own observations have satisfied him. It is stated by Mr. Addison that, when a polygastric animalcule is touched by liquor potassæ, its body bursts, and liberates the so-called stomachs, which are evidently destitute of any structural connexion with it; these corpuscles, when they come under the influence of the same reagent, themselves swell and burst, discharging a number of minute granules; and thus undergoing precisely the same change as that which is effected in the colourless corpuscles of the blood by the alkaline fluid. I cannot doubt that these particles are cells which float in the fluid of the body, and elaborate the materials for its nutrition, in the same manner as do those of the chyle and blood of higher animals.

37. The presence of colourless corpuscles, sometimes to a large amount, in the blood of reptiles, has long been known; since their rounded form and comparatively small size enables them at once to be distinguished from the large oval nucleated red corpuscles of these animals. They bear so strong a resemblance in mammalia, however, to the red corpuscles of the blood, both in size and figure, that their existence, at least to any similar amount, has not been generally recognized. There can be no doubt, however, that they perform as important a function in the blood of mammalia as in that of reptiles; and several facts of the same kind, which have been recently observed, seem to indicate the nature of this function with much probability. It has been (in my opinion at least) satisfactorily ascertained by the observations of Mr. Gulliver, (Philos. Mag. Sept. 1838) and others, that the usual amount of colourless corpuscles in the blood is very much increased at the time when an increase of fibrin is taking place,—that is, in the inflammatory condition; and according to the observations of Remak, Barry, and Addison, the buffy coat usually contains a large number of them. Their increase appears to have some relation with the local inflammatory change; for they are particularly abundant in the blood of inflamed parts, as noticed by Mr. Addison in that drawn by the prick of a needle from a pimple, the base of a boil, the skin in scarlatina, &c.; and they may be seen to accumulate in the vessels of the frog's foot, on the application of stimuli to the part. A corresponding multiplication of the colourless corpuscles takes place in several other instances, in which the formative processes are peculiarly active, and in which the demand for fibrin must therefore be greatly increased. Thus the buffy coat of the blood of pregnant women contains a very large number of them. It is stated by Wagner, (Op. cit. p. 245,) that the number of colourless corpuscles is always remarkably great in the blood of well-fed frogs just caught in the summer season; and that it is very small in those that had been kept long without food, and in those examined during the winter. Their large proportion in the embryo, and gradual decrease as the formative processes become less active, has been already noticed (§ 35).

38. The very facts, therefore, which tend to prove that the *red* corpuscles do not perform the elaboration of the organizable plasma, are equally cogent in favour of the doctrine that this function is to be attributed to the white or *colourless* corpuscles; and this view is borne out by other facts, which indicate that there is a decided relation between the *colourless* corpuscles and the *nutritive* or organic life of the tissues; and a corresponding relation between the *red* corpuscles and their *functional activity.* All observers who have studied the capillary circulation with attention, agree in the statement, that there is a marked difference in the movements of the two classes of corpuscles; the white corpuscles rolling slowly along in the almost motionless layer of plasma which lines the capillaries, whilst the red particles are carried briskly onwards in the centre of the stream. It is difficult to avoid the inference that (to use the words of Mr. Wharton Jones) "there is some reciprocal relation between the colourless corpuscles and the parts outside the vessels, in the process of nutrition." On the other hand, that the presence of the red particles has an important influence in maintaining the excitability of organs, especially the nervous and muscular systems, has long ranked as a physiological truth; and it is fully explained on the principles which Liebig has propounded, with reference to the necessity for oxygen in the active vital operations of the tissues, and the share taken by the red corpuscles in carrying it through the system. Of these views, an account was given in the last Number of this Journal (pp. 499, 521); and it is therefore unnecessary to do more than state here, that Liebig regards a certain disintegration of the tissue as the means of manifesting its vital force, or peculiar property; for this disintegration, the presence of oxygen is requisite; and the oxygen taken in at the lungs is carried through the system chiefly (but not entirely) by the red particles of the blood entering into a chemical combination with their protoxide of iron, which gives place in the systemic capillaries to another,—the oxygen being there set free, and replaced by carbonic acid, which is again exchanged for oxygen in the lungs. That the proportion of red corpuscles in the blood bears a distinct relation to the nervous and muscular energy of the animal, and to the amount of oxygen consumed by it, has long ranked as a physiological fact. An exception might be pointed out in regard to insects, which have no red corpuscles, and yet can display a greater amount of animal energy, and may consume a larger quantity of oxygen in proportion to their size, than beings of any other class whatever. But here, as elsewhere, *exceptio probat regulam;* for the conveyance of oxygen through the tissues is not in them accomplished by the circulating fluid, which has a comparatively sluggish movement, but is effected more directly by the ramifying tracheæ which introduce air into the minutest portions of the structure.

39. It now remains for us to inquire into the origin of the red and colourless corpuscles of the blood. With respect to the first appearance of the former, there is now but little doubt. They are developed from the interior of certain of the cells of the vascular area, the cavities of which cells are fused into each other by the rupture or absorption of their walls, so as to become vessels. That they are subsequently in a continual state of decay and renovation also seems well established; but in regard to the *mode* of this renovation, a question arises, whether they are generated by a change in the chyle and lymph-corpuscles, or whether the new red particles take their origin in the old ones. The former is the opinion of Wagner and many other eminent German physiologists; the latter doctrine was first brought prominently forwards by Dr. Barry, though Leeuwenhoek and other observers after him had entertained it. Although the idea of the origin of the red corpuscles from the chyle-globules has a certain degree of plausibility, with regard to reptiles, in which the chyle-globules have nearly the same diameter with the nuclei of the red particles, it seems totally inapplicable to mammalia, in many of which the red particles are smaller than the chyle globules. Moreover, the appearances which are recorded in support of this doctrine do not show more than that an alteration may take place in the size and form of the chyle-globules, by which their character as nucleated cells becomes more apparent; that these nucleated cells are to become red corpuscles is a matter of supposition only. The author is inclined, by these considerations, as well as from his own observations, to consider Dr. Barry as correct in his statement, that the blood-corpuscles are continually reproducing themselves in the same mode as do other cells; new vesicles being developed from their nuclei, which increase and distend the parent-cell, being at last set free by its rupture or dissolution.

40. Regarding the origin of the colourless corpuscles there is a similar diversity of opinion; by Müller and many other physiologists, they are considered to be identical with the lymph- and chyle-corpuscles; and to this view the author is himself inclined. It is a very strong argument in favour of it that they are of nearly the same size and appearance in all animals that have yet been examined; and that they correspond very closely, not only in these respects, but in the mode in which they are influenced by reagents. There does not seem any greater difference between the colourless corpuscles of the blood and the chyle or lymph-globules, than there is between chyle-globules taken from different parts of the lacteal system. On the other hand, Dr. Barry and Mr. Addison agree in the opinion that the colourless corpuscles are formed from the central portion of the red discs; and they consider them as holding an intermediate position between the true red discs and those greatly altered forms of them which constitute (according to them) the foundations of the tissues, as well as pus- and other globules. It does not appear to the author that the facts stated by either of these observers are sufficient to establish a position so much at variance with known facts. There seems as much difficulty in imagining that the colourless corpuscles of the Frog should have originated from its comparatively *large red* corpuscle,* as that the minute red corpuscle of the Musk Deer should have had its origin in the comparatively large lymph-globule of that interesting animal.† The circumstance that colourless corpuscles exist abundantly in the blood of the fœtus, which has no separate chyle, does not weigh in the least against the doctrine of the identity of the chyle and colourless corpuscles; since the fluid taken up by the omphalo-mesenteric vessels from the surface of the yelk-bag, or from the placental sinuses by the umbilical vessels, stands precisely in the same relation as chyle. The cell-germs of the former are probably furnished by the cells that intervene between the omphalo-mesenteric vessels and the surface of the yelk; those of the latter by the similar layer of cells that covers (according to Mr. Goodsir's observation) the extremities of the absorbent tufts of the umbilical vessels (§ 22).

NUTRITION.

41. The term nutrition may be not improperly limited to the act by which the organizable plasma is converted into solid tissue. This fibrinous plasma is prepared for organization by the assimilating process just described; and if withdrawn from the interior of the living body, it spontaneously passes into a state which presents a definite organization. Hence, the coagulation of fibrin is clearly not the result of its *death,* as was formerly supposed; for this coagulation is the first stage of its organization, when plastic lymph is poured out on a living surface; and even when the process takes place after the complete withdrawal of the fluid from the living body, a fibrous arrangement, as distinct as that which is presented by fibrin coagulated in the living body—up to the time when vessels appear in the newly-forming tissue, is seen in the clot. The fibres may form by their interlacement an areolar tissue; or by their parallel arrangement a distinct membrane. This fact has been noticed by various observers from the time of Haller; but it has been overlooked by many recent physiological writers, and particular attention has lately been directed to it by Mr. Addison and Mr. Gulliver. The former has studied the fibrous arrangement in the act of being formed in the buffy coat; and he has remarked that corpuscles are included in its areolæ, which he believes to be identical with the colourless corpuscles of the blood. Mr. Gulliver has pointed out the same appearance in thin slices of ordinary coagula rendered hard by boiling:‡ and here, too, there are interspersed among the fibres pale corpuscles, which are termed by Mr. Gulliver "organic germs," as well as bodies that resemble nuclei of similar corpuscles. The same arrangement of fibres has been shown by Mr. Gulliver to exist in the false membranes which are produced on inflamed serous surfaces, by the more or less complete organization of fibrinous exudations. In such situations, the fibres are mingled with corpuscles, (termed by other authors "exudation cells,") which seem to be the same "organic germs" in an altered condition.|| These

* Dr. Barry has informed the author that he feels well assured of this transformation; but it cannot be the same with that which produces the colourless corpuscles of the mammalia.

† See Mr. Gulliver's observation, in Willis's Translation of Wagner's Physiology, p. 251, note.

‡ See note by Mr. Gulliver to p. 31 of Gerber's General Anatomy.

|| See Mr. Gulliver's contributions to the Minute Anatomy of Animals, Part iv. Philos. Magazine, Oct. 1842.

corpuscles may form a larger or smaller proportion of the exudation; if they are merely scattered amongst the fibres of the coagulum, and the chief part of it be composed of hardened fibrin, the membrane will be tough; but if the exudation be chiefly composed of these corpuscles, with but a small amount of fibres between them, the membrane will be quite friable, and will approach towards the character of a purulent exudation.

42. The fibrous aggregation of the particles of fibrin seems, therefore, to be the real process of coagulation, whether upon a living or a dead surface. By Dr. Barry it is imagined that the appearance of fibres in the coagulum is due to the rupture of some of the blood-discs in which he believes fibres to be generated, and the consequent escape of the latter; but it seems to be forgotten by him that fibrin will coagulate without any blood-discs,— as when the latter are separated by filtration, according to Müller's celebrated experiment upon frog's blood,—or when the chyle, in which no blood-discs are to be found except by an accidental admixture, is withdrawn from the thoracic duct. Moreover, as Mr. Gulliver's figures, (Gerber's Anatomy, Figs. 244-6,) all copied accurately from nature, clearly show, a small portion of coagulated fibrin contains a far larger number of fibres, than we can imagine to be contained in the number of blood-discs that would fill the same space. The author has lately discovered a very interesting example of a membrane composed almost entirely of matted fibres, which so strongly resembles the delineations of fibrous coagula given by Mr. Gulliver, that he cannot but believe in the identity of the process by which they are produced. This is the membrane inclosing the white of the egg and forming the animal basis of the shell. If the shell be treated with dilute acid a tough membrane remains, exactly resembling that which lines it; and if the hen has not been supplied with lime there is no difference between the two membranes even without the action of acid on the outer one. Each of these membranes consists of numerous laminæ of most beautifully matted fibres intermixed with round bodies exactly resembling exudation cells. It is in the interstices of these fibres that the calcareous particles are deposited which give density to the shell; these membranes are formed around the albumen which is deposited upon the surface of the ovary during its passage along the oviduct, from the interior of which the fibrinous exudation must take place. All these facts clearly indicate, that for the reparations of injuries, *inflammation* is not an essential change; since the ordinary fibrin which is continually being applied to the purposes of nutrition, is capable of passing spontaneously into the organized condition, and thus of forming a regular tissue, for the more complete organization of which, nothing is required but the extension of vessels into it from the subjaceut surface. Thus a strong confirmation is afforded to Dr. Macartney's doctrine, that the reparation of injuries is best effected by a process resembling the ordinary nutrition of the tissues; and that our therapeutic efforts should be directed to promote this, and to keep down inflammation.

43. The doctrine of Schwann respecting the development of fibrous as well as other tissues from cells appears to require some modification, since we thus see fibres produced by the simple consolidation of the plasma without an intervening development of cells. Yet if the preceding doctrines be correct, the agency of cells is still required for their production, though in an entirely different mode; since the fibrinous plasma in which the fibres originate is itself elaborated by the cells floating in the circulating fluid. The same remark applies to the other instances in which a tissue appears to be produced without the intervention of cells. Thus the essential part of a mucous membrane, according to Bowman and Goodsir, is a delicate structureless lamella; in the production of which cells appear to have no concern. A similar homogeneous membrane forms the lining of the arteries; but the membrane contains minute particles, which appear to be the germs of the epithelium-cells that are to be developed on its surface. A continual supply of such germs must be required where the epithelium-cells are being constantly thrown off, as is the case with those of the stomach and intestinal canal and with secreting surfaces in general. These germs or reproductive granules have probably been prepared by those *assimilating cells,* the influence of which on the plasma has prepared it to pass into the condition of membrane.

44. There can be no doubt, however, that the function of the plasma or liquor sanguinis is, in general, to supply the material for the nutrition of the previously-formed tissues,—that is, for the reparation of their waste, by the production of new tissues like their own. As to the mode in which this is accomplished there is much that is still very obscure, notwithstanding the recent vast increase in the amount of knowledge on the subject. It is certain that a large proportion of the tissues are produced in the embryo, from the cells of which alone it is composed at an early period; these cells undergo various kinds of metamorphoses, the nature of which will be detailed in a future report. But, according to Dr. Barry, many of these tissues which make their appearance in the midst of others,—the crystalline lens for example,—originate in cells which he believes that he can trace back to the *red* corpuscles of the blood. Regarding the validity of this statement, materials are yet wanting for a positive decision; for those which Dr. Barry's paper contains can scarcely be regarded as decisive. The origin of tissues from the *colourless* corpuscles appears to be a much more probable supposition. The "exudation cells" which are found in the lymph effused on cut surfaces, and the pus-globules into which these may be converted by the action of air or other causes, bear so strong a resemblance to the colourless corpuscles, that it is difficult to refer them to any other origin. This, indeed, is the view of their nature entertained by Barry and Addison; and it is only by referring the colourless corpuscle itself to the red particle that they can trace back the greater number of tissues to the latter; the colourless corpuscles being in their view an intermediate stage of development between the ordinary red particles and the first cells of newly-forming tissue. The determination of this last question is a work that cannot be accomplished, except by an extensive series of observations carried on through a great range of species.

45. But even supposing that the *origin* of any mass of tissue should be traced back to either kind of the cells that are floating about in the blood, we are not thence to decide that the *continued nutrition* of the tissue is performed in the same manner. The muscular fibre once formed, may be able, for anything that we know to the contrary, to produce the germs of other fibres, by the materials elaborated from the blood, without any direct supply of cells or fibres from it. We *know* this to be the case in regard to cartilage, the cells of which are continually undergoing increase by a process of multiplication exactly conformable to that which takes place in the early state of the embryo; and we know that this tissue is not supplied with blood in any other way than by the transudation of the plasma through its substance. Again, on the surface of mucous membranes there is a continual new development of epithelial cells; these can scarcely be altered blood-corpuscles, as Barry and Addison consider them to be, since bodies of such large size could not make their way through the basement membrane without sensible pores, which certainly do not exist. There seems little doubt that the rapid renewal of the epithelium-cells, which is continually taking place on many of the mucous surfaces, is due to the development of germs contained in the basement membrane at the expense of the fluid brought to its attached surface by the vessels ramifying beneath it. That Dr. Barry and Mr. Addison should see a strong resemblance between blood-corpuscles and incipient epithelium-cells is not surprising, when it is considered how much alike *all* cells are in some or other of their stages of production; and when the *impossibility* (for such it clearly appears) of this transformation is considered, much doubt is necessarily cast on the validity of the other inferences of those observers. If *similarity* alone is to be taken as a proof of *identity*, then the identity of the chyle and lymph-globules with the colourless corpuscles of the blood is a necessary inference.

46. The foregoing observations are not intended to express any decided opinions on the subject of the formation of the tissues; since the whole question appears to the author to be at present *sub judice*. In his own mind, however, there is a decided preponderance of evidence in favour of the opinion, that the perpetual reproduction of tissue which constitutes the act of Nutrition, is due to the development of cell-germs in the tissues themselves, at the expense of the fibrin of the blood; and that the use of the *white* corpuscles (of which the analogues are found in the circulating fluid of all animals) is to elaborate that fibrin; whilst the function of the *red* corpuscles (whose office must be of a more special nature, since they are only to be found in vertebrata,) is to serve as the carriers of oxygen and carbonic acid. But the determination of it has no importance in regard to the principle which is developed in this report. Whether or not it be true that the tissues have their origin in the red corpuscles of the blood, as Dr. Barry maintains, they are developed by the agency of cells; and these cells are descended, more or less remotely, from those of which the foundations were deposited in the germinal vesicle by the act of fecundation. The doctrine of cell-life is as true, therefore, when applied to animal as to vegetable nutrition.

### SECRETION.

47. There can scarcely be a more beautiful illustration of the doctrine, that physiology is as capable as any other science of being reduced to general principles, and that these principles must, if valid, be of *universal* application, than the fact that the process of secretion is performed, in animals as in plants, by the agency of *cells;* and that however complex the structure of the secreting organ, these simple bodies constitute its really operative part. The progress of comparative anatomy had shown that neither its form nor its internal arrangement could have any essential connexion with the nature of its product; since even those glands (the liver and the kidney for example) in which there is the greatest peculiarity of structure, make their first appearance in the simplest possible form. Still something was wanting to prove that the structural elements immediately concerned are in all instances the same; and there seemed no analogy whatever between the secreting *membrane* of the animal and the secreting *cell* of the plant. The doctrine was first propounded by Purkinje, adopted and extended by Henle, and fully confirmed by the interesting researches of Goodsir and Bowman, that true *secretion*—that is, the elaboration from the blood of certain of its solid contents, which previously existed there in a form more or less different from that in which they afterwards present themselves—is always performed by the intervention of cells; which, as a part of their regular vital actions, withdraw these ingredients from the blood and afterwards set them free by their own rupture or dissolution. The process is thus strictly analogous to that of nutrition; since *every* cell, in the progress of its development, forms certain peculiar products out of the alimentary materials supplied to it; and just as the cells at the extremities of the the villi select from the chyme the nutritive portion which is to be introduced into the absorbent vessels, so do the cells that line the secreting tubuli select from the blood the effete particles it is *their* peculiar province to assimilate, and discharge them into the canals by which they will be carried out of the system. As Mr. Goodsir very justly remarks, "There are not, as has hitherto been supposed, two vital processes going on at the same time in the gland, growth and secretion; but only one, viz. growth. The only difference between this kind of growth and that which occurs in other organs is, that a portion of the product is, from the anatomical condition of the part, thrown out of the system." (Transactions of the Royal Society of Edinburgh, vol. xv., p. 302.)

48. There cannot be a better illustration of this view than the nature of *fat,* the production of which is exactly the intermediate link required to connect the two processes. The adipose tissue consists of cells, by the action of which the fatty matter is elaborated from the blood; instead, however, of being thrown out of the system, it remains stored up in their cavities until it is required for use within the body; and it must then be taken into the circulation by a process resembling the first absorption of aliment. Now a certain proportion of fatty matter is normally found in the secreting cells of the liver, and this quantity may be very much increased, as Mr. Bowman has shown, especially in diseases which obstruct the pulmonary circulation. The fat elaborated by *these* cells is destined to be thrown off from the system; and thus we perceive how much the anatomical position of the cells has to do with the result. Mr. Gulliver has communicated to me an interesting observation on the state of the secreting cells of the liver in jaundice, as witnessed by him in two cases. They were found to contain an unusual quantity of biliary matter, (easily distinguished by its colour) which was collected chiefly around the nuclei, but was also scattered throughout the cell. Some of the cells were nearly opaque from the great quantity of biliary matter contained in them. In healthy cells of the liver the same appearance is not seen; for they are of a light brown colour and almost transparent. It would be interesting to examine the state of the hepatic cells, in those cases in which there is not a retention but a suppression of the secretion. Another interesting example of abnormal secretion has been mentioned to me by the same industrious observer. On examining the so-called *piogenic membrane* lining a chronic abscess, he found it to consist of pus-like globules, strongly resembling the colourless corpuscles of the blood, with minuter molecules, and united by fibrinous fibrils, altogether very much like the false membrane which he has depicted as lining a tuberculous cavity (Philosophical Magazine, Nov. 1842). The contents of the abscess were common pus, mixed with a considerable quantity of fibrinous matter in masses of variable size,—the concrete or lardaceous pus of the French. The *secretion* of the purulent fluid, therefore, takes place in such instances from a membranous surface chiefly composed of cells analogous to those which are present in the *liquor puris* in greater or less amount.

49. The production of cells, as an integral part of the normal process of secretion, has been demonstrated by Mr. Goodsir in a considerable variety of instances (Figs. 11-13); and he has further shown that what is ordinarily termed an *acinus* is nothing more than a parent-cell filled with progeny. (Fig. 14.) This statement may also, as appears from late researches, be applied to the lungs, in which the air-tubes do not terminate, as maintained by Reissessen and his followers, in single dilated cæca, but open into a system of communicating beaded canals, forming a kind of acinus. These beaded canals are evidently composed of cells partly fused together; and by the comparison of their state in animals of different ages, it seems that they are all developed from the cell in which the air-tube terminates, and that they continue to increase in number from the period of birth to adult age. The fact already stated, respecting the function of the red corpuscles of the blood and their connexion with the respiratory function, supplies the required proof that respiration takes place through the medium of cells. But it may be questioned whether such agency is necessary where, as in insects, the air comes into more immediate contact with the blood; the change being of a chemico-physical character, and not truly vital.

50. The structure of the testes also, and the nature of their product, have an interesting connexion with the structure and functions of the ordinary secreting organs. It was ascertained by Wagner that the most characteristic portion of the testes, throughout the animal kingdom, are the cells in which the spermatozoa are generated. These cells are found among the lower animals to lie in the midst of other tissues, and to set free their products by rupture, just as do their secreting cells. In the higher animals the process cannot be so well observed, since the cells lie in a testis of more complex construction, which seems destined to form some other secretion; but still it takes place in a manner essentially the same; and thus the proof is complete, that in the animal, as in the plant, the organic functions are all performed through the agency of cells.

---

EXPLANATION OF THE PLATE.

Fig. 1. An ovum prepared for fecundation, from the ovarium of a Rabbit in heat. The germinal vesicle, *c*, contains numerous concentric layers of cells, which have been successively formed at the margin of the germinal spot; the altered state of this is seen at *b*. The germinal vesicle is surrounded by a mass of cells, *d*, in each of which a corresponding process is taking place; these are indistinct, and appear to be undergoing liquefaction, beneath the zona pellucida, *f*. (§ 12.)

Fig. 2. Plan of the structure of one of the parent-cells of the substance surrounding the germinal vesicle; this is seen to contain several concentric layers of

1
2
3
4
5
6
7
8
9
10
11
12
13
14
a
b
c
d
e
f
g
cho young cells, in the outermost and oldest of which a similar set of changes is going on. (§ 12)

Fig. 3. A fecundated ovarian ovum of the Rabbit, five hours and a half after copulation. The germinal vesicle, *c*, filled with cells, appeared to be returning to the centre of the ovum. A distinct cleft appears in the part of the zona pellucida, *f*, just above the spot it had quitted. (§ 13.)

Fig. 4. A fecundated ovum twenty-four hours and a half, from the fallopian tube. Two new cells, *b*, are now seen in the midst of those which previously filled the germinal vesicle, and which are now beginning to liquefy; all the cells of the yelk have undergone liquefaction, except two marked *d*. (§ 13.)

Fig. 5. An ovum of twenty-four hours and a half from the fallopian tube, more advanced than the last. The twin cells, *b*, now occupy nearly its whole interior, and are filled with new cells, of which two are destined permanently to replace each. All the previously formed cells have undergone liquefaction. (§ 13.)

Fig. 6. Cells from the group which originates in the twin cells; in one of them are represented the two cells that will succeed it, themselves beginning to undergo the same series of changes. (§ 17.)

Fig. 7. An ovum of sixty two hours, from the uterus, showing the "mulberry mass" of cells which has originated from the twin cells, *b*, of Figs. 4 and 5. The ovum is now enveloped in the chorion, between which and the zona pellucida there is a considerable amount of fluid. (§ 17.)

Fig. 8. An ovum of 102½ hours from the uterus, showing (on a smaller scale) a much later stage in the development of the germinal structure. The mulberry mass, *b*, has reached the side of the ovum; and such a change in its arrangement has taken place, as to bring into view the central cell, *a*. Within this is seen its large annular nucleus, surrounded by the pellucid area. The margin of the mulberry mass has spread itself over the interior of the zona pellucida, forming the membranous expansion, *c*, which is ordinarily known as the serous layer of the germinal membrane. (§ 18.)

The preceding figures are all copied from the delineations accompanying Dr. Barry's Embryological Researches. The succeeding are from Mr. Goodsir's.

Fig. 9. Extremity of a villus during the absorption of chyle; the absorbent vesicles at its extremity are distended with chyle; and the trunks of its lacteals are seen through its coats. (§ 22.)

Fig. 10. Two follicles from the liver of the crab. The colourless germinal spot is at the blind extremity of the follicle. The secreting cells become distended with bile and oil as they recede from the germinal spot.

Fig. 11. Secreting cells from the liver of the Limpet. The bile is contained within the secondary cells, which occupy part of the cavity of the parent-cell.

Fig. 12. Secreting cells from the kidney of the Snail. The contained secretion is dead white, and presents a chalky appearance.

Fig. 13. Secreting cells from the mamma of the Bitch, containing milk-globules.

Fig. 14. Progressive stages in the development of the testis of Squalus cornubicus (Porbeagle); *a*, portion of duct, with nucleated cells attached to its walls; *b*, one of these cells more developed and containing young cells within it, now altogether constituting an acinus; *c* and *d*, more advanced stages of the primary cell; *e*, secondary cells beginning to assume a cylindrical form; *f*, this change completed; *g*, secondary cells, one in its first state with a composite nucleus, another elongated into the cylindrical form and containing spermatozoa, which are also represented free. (§ 49.)

For a fuller explanation of Fig 14, see p. 565 of the last Volume.

## Figures and Tables

**Figure f1:**